# Accelerated weight gain, prematurity, and the risk of childhood obesity: A meta-analysis and systematic review

**DOI:** 10.1371/journal.pone.0232238

**Published:** 2020-05-05

**Authors:** Mei-Chen Ou-Yang, Yao Sun, Melissa Liebowitz, Chih-Cheng Chen, Min-Lin Fang, Weiwei Dai, Tang-Wei Chuang, Jyu-Lin Chen

**Affiliations:** 1 Department of Pediatrics, Kaohsiung Chang Gung Memorial Hospital, Chang Gung University, College of Medicine, Kaohsiung, Taiwan; 2 Department of Pediatrics, University of California, San Francisco, California, United States of America; 3 Medicine Library and Center for Knowledge Management, University of California, San Francisco, California, United States of America; 4 Xiangya Hospital, Central South University, Changsha, P.R. China; 5 Division of Hepatogastroenterology, Department of Internal Medicine, Chi Mei Medical Center, Liouying, Taiwan; 6 School of Nursing, University of California, San Francisco, California, United States of America; University of South Florida, UNITED STATES

## Abstract

The purpose of this systematic review and meta-analysis of the literature was to analyze and evaluate the impact of prematurity and accelerated weight gain on the risk of childhood and adolescent obesity. CINAHL, Embase, PubMed, and Web of Science databases were searched until December 2019 which yielded 19 studies with a total of 169,439 children enrolled were systematically reviewed. The results revealed that preterm infants had a greater likelihood of childhood obesity (defined as BMI ≥95^th^ percentile for age-sex), than term infants (OR = 1.19, 95% CI [1.13, 1.26]). However, no difference of childhood obesity was found between “small for gestational age”(SGA) and “appropriate for gestational age”(AGA) among preterms. Accelerated weight gain (defined as weight gain velocity during first two years after birth) significantly increased the likelihood of subsequent childhood obesity among preterms (aOR = 1.87, 95% CI [1.57, 2.231]). In conclusion, accelerated weight gain at infancy among preterm children may be a critical contributor to obesity in later life. Establishing optimal growth trajectories and timely referral to health care providers may be of clinical importance.

## Introduction

The rapid increase of obesity in children and adolescents, a worrisome trend, has emerged as a public health issue in both developed and developing countries [1–3]. Childhood obesity is associated with increased morbidity and mortality in adulthood [4, 5]. Several factors have been shown to increase the risk of childhood obesity, including prematurity, small or large for gestational age (GA), small or large birth weight, and a family history of obesity-related diseases [6–10]. The World Health Organization (WHO) defines prematurity as “born alive before 37 weeks of pregnancy.” According to the WHO, the prevalence of preterm birth ranges from 5% to 18%, including approximately 12% in lower-income countries and 9% in higher-income countries. Recent technological advances in neonatal care have led to significant increases in the survival rate of preterm infants. However, several health issues are associated with prematurity, including metabolic diseases and obesity [11–14].

Weight gain in preterm infants is used as an indicator of growth and a criterion for hospital discharge [15]. Several studies have reported that “catch-up growth” and weight gain in preterm infants during the critical postnatal period (early neonatal growth, postdischarge growth in early infancy) are associated with better cognitive outcomes [16–20]. However, other studies have reported that nutritional supplements that accelerate catch-up growth increase the risk of future obesity and lead to metabolic syndrome [21–23]. Environmental influences may also lead to obesity and metabolic syndrome via epigenetic programming in preterm infants [24]. The Barker hypothesis and thrifty gene hypothesis suggest that susceptibility to chronic diseases in adulthood can be caused by exposure during the prenatal and perinatal periods that increase the likelihood that preterm infants will store extra fat and energy [25]. However, associations between preterm birth and rapid weight gain in preterm infants and the risk of obesity have not been systematically analyzed.

Childhood obesity has been reported to increase the risk of adulthood obesity [26] and to be associated with more chronic conditions in adulthood [27]. It has been proposed that certain critical periods in infancy, such as GA and early development, are associated with an increased risk of childhood obesity [23, 27]. Earlier population-based studies and systematic reviews have identified a moderate association between birth weight and later risk of obesity in full-term infants with a low birth weight (< 2500 g) and high birth weight (> 4000 g) [10, 28–31]. Although premature birth is a risk factor for obesity, few researchers have examined the association between premature birth, defined by GA, and the risk of obesity in later life [32–34]. Preterm infants and term infants experience postnatal growth trajectories and the complexity of environmental exposure differently [35]. However, the difference in the risk of obesity between preterm and term infants has not been examined. Moreover, although differences in the risk of obesity between appropriate for gestational age (AGA) and small for gestational age (SGA) preterm infants has been studied, it has yet to be systematically evaluated. To address this gap in the literature, we systematically examined the effects of preterm birth and rapid weight gain in infancy on the risk of childhood obesity. Understanding these effects of preterm birth and rapid weight gain on the risk of childhood obesity in preterm infants can provide clinical guidance on developing appropriate weight gain and growth trajectories for this high-risk population.

The aim of this meta-analysis was to systematically analyze and evaluate the influence of prematurity and rapid weight gain on the associations between prematurity and the risk of obesity in childhood and adolescence. Childhood obesity is defined as body mass index(BMI) percentile greater than 95^th^ percentile for age-sex. We designed this study to answer these three questions:

Research question 1: What is the impact of GA on the risk of childhood obesity between term and preterm infants?
Research question 2: In preterm infant, is SGA associated with greater risk of childhood obesity compared to AGA infants?
Research question 3: Does accelerated weight gain in preterm infants increase the risk of childhood obesity?

## Methods

This study was conducted in accordance with the PRISMA (Preferred Reporting Items for Systematic Reviews and Meta-Analyses) guidelines [36].

### Eligibility criteria

This systematic review and meta-analysis included studies that reported the association between preterm birth (≤ 37 weeks GA) and the subsequent risk of obesity in childhood and adolescence. Studies were eligible if they were published in English from inception to December 16, 2019, which focused on preterm infants and reported at least one type of outcome measures for obesity such as BMI, body fat, or waist circumference between 3 to 18 years of age. Retrospective or prospective observational studies were eligible to be included in this review. The approach of English language restriction was justified for not introducing any additional bias in the previous study [37]. Studies that focused on adulthood obesity only, conference abstracts, and review studies, were excluded.

### Information source and search strategy

With the assistance of an expert librarian (M.L.F.), a comprehensive search of the CINAHL, Embase, PubMed, and Web of Science databases from inception to December 16, 2019, was performed. The librarian assisted in developing search terms and subsequently conducted the search itself using the following keywords:infant, premature [mh] OR infant, extremely premature [mh] OR premature birth [mh] OR infant, low birth weight [mh]) OR (preterm* [tiab] OR premature [tiab] OR premature birth* [tiab] OR low birth weight [tiab])AND (obesity [mh] OR obesity [tiab] OR pediatric obesity [mh] OR "pediatric obesity" [tiab] OR "paediatric obesity" [tiab]). Search filters were: humans, abstract available and English language. We also cross-referenced and manually searched the bibliographies of pertinent studies.

### Study records data and selection

Two independent investigators (M.C.O.Y. and J.L.C.) evaluated the studies for eligibility. Discrepancies or disagreements on which studies to include were resolved through discussion and consensus. When no agreement could be reached between the two investigators, a third investigator (Y.S.) was asked to decide eligibility.

### Data extraction

Study data were extracted including general information, surname of the first author, the year of publication, study population, type of obesity outcome and definition, study design timing of outcome evaluation, statistical method, and adjusted odds ratios or mean differences between the study groups When adjusted odds ratios or risk ratios were not reported by the studies included, the event numbers or event rates were extracted, then crude ratios were calculated by our statistician.

### Quality assessment

Two members of the research team (M.C.O.Y and J.L.C.) independently reviewed each article twice for methodological quality using a quality rating tool previously validated [38]. This a toolwas used to assess four domains with 11 questions. Each satisfactory answer received one point, resulting in a maximum score of 11 Eachstudy was rated as low (0–5), moderate (6–7) or high quality (> 8). Inconsistencies in scoring were resolved by consensus.

### Data synthesis and analysis

A meta-analysis was conducted to calculate pooled odds ratios (ORs) with 95% confidence intervals (CIs) for the effects of factors of interest on childhood obesity. Standardized mean differences (SMD) with 95% CIs werecalculated based on the original mean, standard deviation(SD) and sample size of eligible studies to estimate the association between childhood fat mass index and preterm status We applied a random-effects model because we could not assume those study settings were identical; this model provided a more conservative result than a fixed model. Forest plots were used to graphically depict individual and pooled effect sizes. We used two methods to assess heterogeneity: Cochran’s Q Test, which is considered to be statistically significant for heterogeneity if the *p* value is < 0.1, and I^2^ statistics, wherein values 30% to 60% and 60% to 90% suggests moderate and substantial heterogeneity, respectively. We used STATA version 14 software (Stat Corp, College Station, TX, USA) for the meta-analysis.

## Results

### Overview of studies in the systematic review and meta-analysis

**Fig 1:**
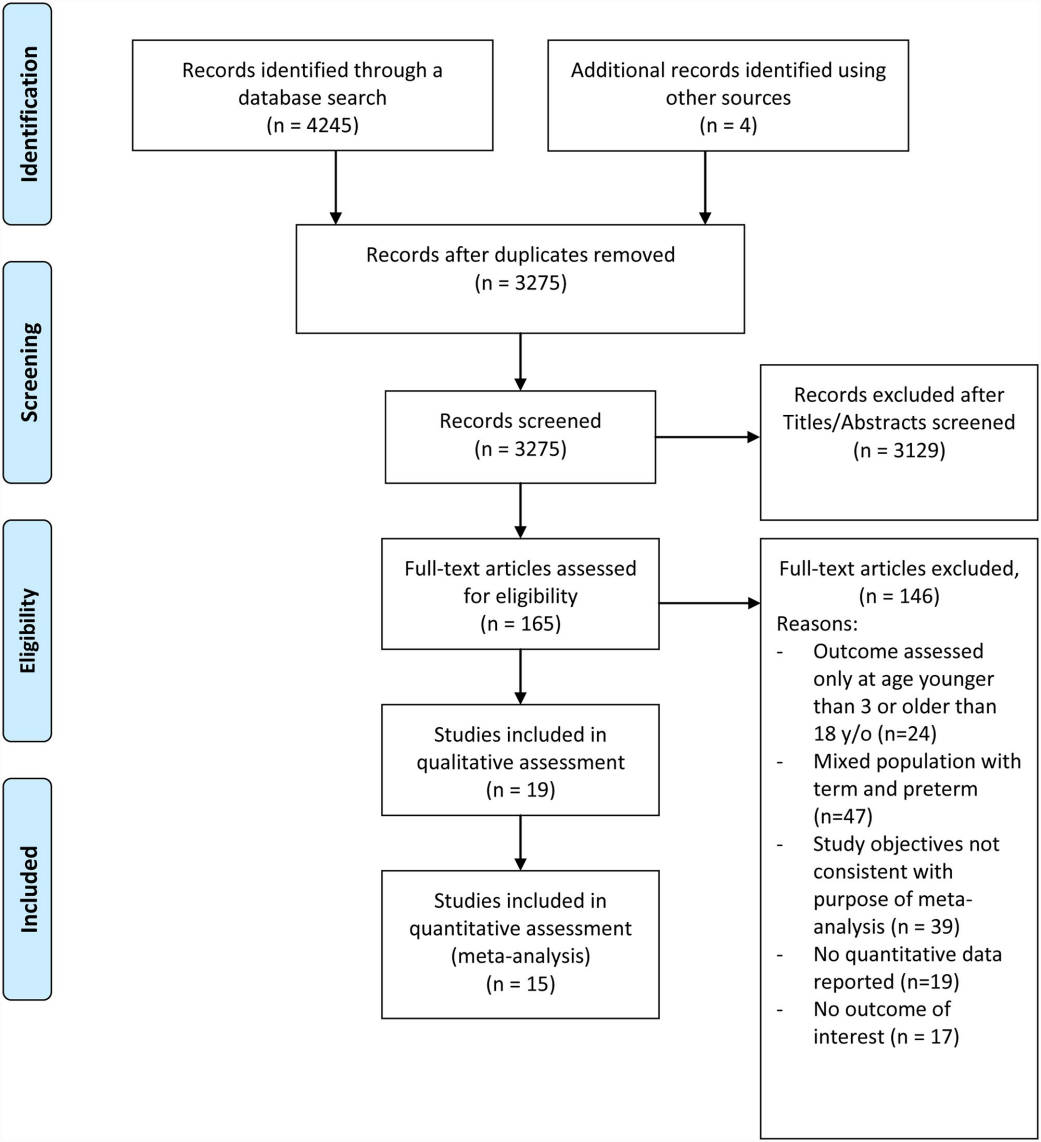
PRISMA flow diagram of studies included.

Our search yielded 4,249 articles, of which 3275 non-duplicate articles were screened (Fig 1). Of the 165 articles that underwent full-text review, 146 were excluded and 19 studies(6 retrospective and 13 prospective) with totally169,439 children enrolled met the eligibility criteria and were included in this review. The characteristics of the studies included in the review and their criteria for defining obesity are summarized in Table 1.(Table 1).

**Table 1:** Characteristics of the studies included.

First author, year	Study design/country	Total number of children	Number of preterm, term, SGA / AGA, and gestational age	Age of OBE evaluation (years)	Indices for OBE	Main finding
Alves, 2016 [39]	Retrospective, Brazil	134	67 Preterm (mean GA 33.2 wk); SGA: 30; 67 Term	10–13	BMI >97th percentile;	OBE, preterm vs. term: 6 (14.3%) vs. 6 (12.2%), p = 1.00
Belfort 2013 [52]	Retrospective, USA	945	945 Preterm (median GA 33.26 wk); SGA: 327	8–18	Z-score for corrected age	Associations with overweight/OBE:
						BMI z-score change at 4 months: adjusted OR = 1.36 (1,14–1.62)
						BMI z-score change at 4–12 months: adjusted OR = 1.66 (1.33, 2.06)
						BMI z-score change at 12–18 months: adjusted OR = 2.00 (1.53, 2.61)
Casey, 2012 [49]	Prospective USA	686	686 Preterm (mean GA 33 wk)	8	BMI for age-sex ≥ 95^th^ percentile	Associations with OBE:
						Rapid weight gain (weight gain velocity 100g/mo, from birth to 12-mo): adjusted OR = 2.7, 95% CI [1.9, 3.9]
						SGA status: adjusted OR = 0.6, 95% CI [0.26, 1.5].
Darendelil, 2008 [46]	Prospective, Turkey	179	93 Preterm (≤ 37 wk); AGA / SGA: 63 / 30; 86 Term; AGA / SGA: 44 / 42	4.7	Fat mass index	Preterm AGA vs term AGA had similar fat mass index and trunk fat index. Fat mass index: 3.6±0.4 vs. 2.7±0.1; trunk fat index:1.5±0.2 vs. 1.1± 0.1.
						Preterm SGA vs. preterm AGA had similar fat mass index and trunk fat index. (2.9±0.5 vs. 3.6±0.4; 1.2±0.3 vs. 1.5±0.2).
Embleton, 2016 [21]	Prospective, UK	98	98 Preterm (mean GA 30.8 wk)	11.5	% Body Fat; Fat mass index; Waist circumference	Adolescent height and weight SDS did not differ between rapid weight gain or not (0.01±0.92 and 0.3±1.2, respectively). (Rapid weight gain: weight z score change > 0.67 from term to 12 wk, n = 24)
						Rapid weight gain after 1 year of age was associated with subsequent higher % fat mass, fat mass index and waist circumference (coefficient: 5.03, 95% CI [3.74, 6.32]; 1.74, 95% CI [1.35, 2.13]; 5.89, 95% CI [4.28, 7.50]).
Gaskins, 2010 [9]	Prospective, USA	312	312 Preterm (≤ 32 wk:115, 32–36 wk: 197); SGA/AGA: 67/245	11	BMI for age-sex >95^th^ percentile	Associations with OBE:
						Rapid weight gain (weight gain velocity g/mo, from birth to 12-mo): adjusted OR = 2.69; 95% CI [1.80, 4.00]
						SGA status: adjusted OR = 2.28; 95% CI [0.95, 5.46].
Gianni, 2008[42]	Prospective, Italy	95	45 Preterm (<34 wk; mean GA 30.5±1.9 wk); 40 Term (mean GA 39.1±1.3 wk)	4.8–6.6	Fat mass index	Fat mass index (kg/m^2^) was lower in preterm (2.76±1.16 vs. 3.76±1.58, *p* < 0.05); Trunk fat index (kg/m^2^) was not significant (PT vs. FT, 0.94±0.73 vs. 1.18±0.72); Preterm SGA positively affected trunk fat mass content (r^2^ = 0.37, *p* < 0.05).
Gianni, 2015 [43]	Prospective, Italy	124	63 Preterm (< 32 wk); 61 Term	5	% Body fat; Fat mass index	% Body fat and fat mass index were similar in both groups. % Body fat: 20.4±5 (boy PT) vs 17.7 ± 5.5 (boy FT); 20.5±5.1 (girl PT) vs. 23.1±4.7 (girl FT).
						Fat mass index: 3.1±0.9 (boy PT) vs. 2.9±0.1 (boy FT); 3.2±1.1 (girl PT) vs. 3.7±1.2 (girl FT).
Hack, 2011 [40]; 2014 [87]	Prospective, USA	259	146 Preterm (mean GA 26.5 ± 2); 113 Term	14	BMI for age-sex ≥ 95^th^ percentile	OBE, preterm vs. term: 28 vs. 23
Hui, 2015[48]	Prospective, Hong Kong	7169	295 Preterm (mean GA 35.4 wk); 6874 Term	14	BMI z score; WHR z-score; WHtR z-score;	Preterm had greater WHR z-score (β = 0.16, 95% CI [.03, 0.29]) and WHtR z-score (β = 0.27, 95% CI [0.14, 0.40]) compared with term infants.
Huke, 2013[44]	Retrospective, Germany	236	116 Preterm (≤ 33 wk; mean GA 29.8 ± 2.6 wk); 120 Term	5–7	% Body fat; Fat mass index; Waist-hip circumferences; Adipose tissue by MRI	Waist-hip circumferences similar in both group (0.97 vs. 0.96 in preterm and term group).
						% Body fat, fat mass index were lower than term group (18% vs. 21%, *p* = 0.0022; 2.82 ± 1.4 vs. 3.36 ± 1.32 kg/m2, *p* = 0.028)
						TAAT(cm^3^): preterm vs. term: 72.1 ± 33.8 vs.87 ± 55.8, *p* = 0.04%
						IAAT (%): preterm vs. term: 30 ± 9 vs. 28 ± 9, *p* = 0.23.
Mardones, 2008 [41]	Retrospective, Chile	153536	17574 Preterm	6–8	BMI for age-sex ≥ 95^th^ percentile	OBE, Preterm vs. term: 17.53% vs. 18.05%, p = 0.088
			135962 Term			
Ramirez-Velez, 2017 [14]	Retrospective, Colombia	2510	1092 Preterm (< 37 wk); SGA / AGA: 249 / 843; 1418 Term; SGA / AGA: 260 / 1158	11–14 Mean age:13.2	BMI for age-sex ≥ 95^th^ percentile	Risks of OBE were not significant different in preterm vs term, adjusted OR = 1.373, 95%CI [0.93, 2.02]) (preterm vs. term: n = 54 vs. 55)
						SGA status was significantly associated with OBE, adjusted OR = 1.07, 95% CI [0.42, 1.03])
Vasylyeva, 2013 [11]	Retrospective, USA	147	147 Preterm (≤ 37 wk); SGA/AGA: 23/124	10–20	BMI for age-sex ≥ 95^th^ percentile	Associations with OBE: SGA status: adjusted OR = 0.47, 95% CI [0.13, 1.69].
Vohr, 2018 [50]	Prospective, USA	388	388 Preterm	6–7	BMI for age-sex ≥ 95^th^ percentile	Rapid weight gain (weight gain velocity kg/yr from birth to 18–22 months) was not significantly associated with OBE, adjusted RR = 1.23, % 95CI [0.95, 1.60], p<0.123
Willemsen, 2008 [47]	Retrospective, Netherlands	144	51 Preterm (< 36 wk, all SGA); 93 Term (all SGA)	6.8	% Body fat SDS; Trunk fat/total fat	PT SGA had lower body fat SDS than term SGA (-1.2 (0.8) vs. -0.6(0.9), Similar trunk fat/total fat 0.33 (0.05) vs. 0.34 (0.05).
Wood, 2018 [51]	Prospective, USA	743	743 Preterm	10	BMI for age-sex ≥ 95^th^ percentile	Rapid weight gain (top quartile weight gain from birth to 12 months) was significantly associated with OBE, adjusted OR = 2.4, 95% CI [1.5–3.9].
Zanini, 2014 [45]	Prospective, Brasil	1734	416 Preterm (< 37 wk)	6.7	% Body fat; Fat mass index	Lower % body fat and fat mass index were found in preterm.
			1318 Term			
						% Body fat: PT vs. FT: 17.61±0.53 vs. 21.05±0.16; fat mass index: PT (*n* = 403) vs. FT (*n* = 2643), 3.18±0.14 vs. 3.83±0.05 [88].

**Abbreviations**: *n*, sample size; SGA, small for gestational age; AGA, appropriate for gestational age; BMI, body mass index; OBE, obesity; OR, odds ratio; CI, confidence interval; GA, gestational age; f/u, follow-up; y/o, year old; DEXA, dual-energy x-rayabsorptiometry; SDS, standard deviation score; BIA, bioelectrical impedance analysis; MRI, magnetic resonance imaging; TAAT, total abdominal adipose tissue; PT, preterm; FT, full term; %IAAT, intra-abdominal adipose tissue/total abdominal adipose tissue; WHR, waist-hip ratio; WHtR, waist-height ratio; wk, week

### Quality assessment summary

**Fig 2:**
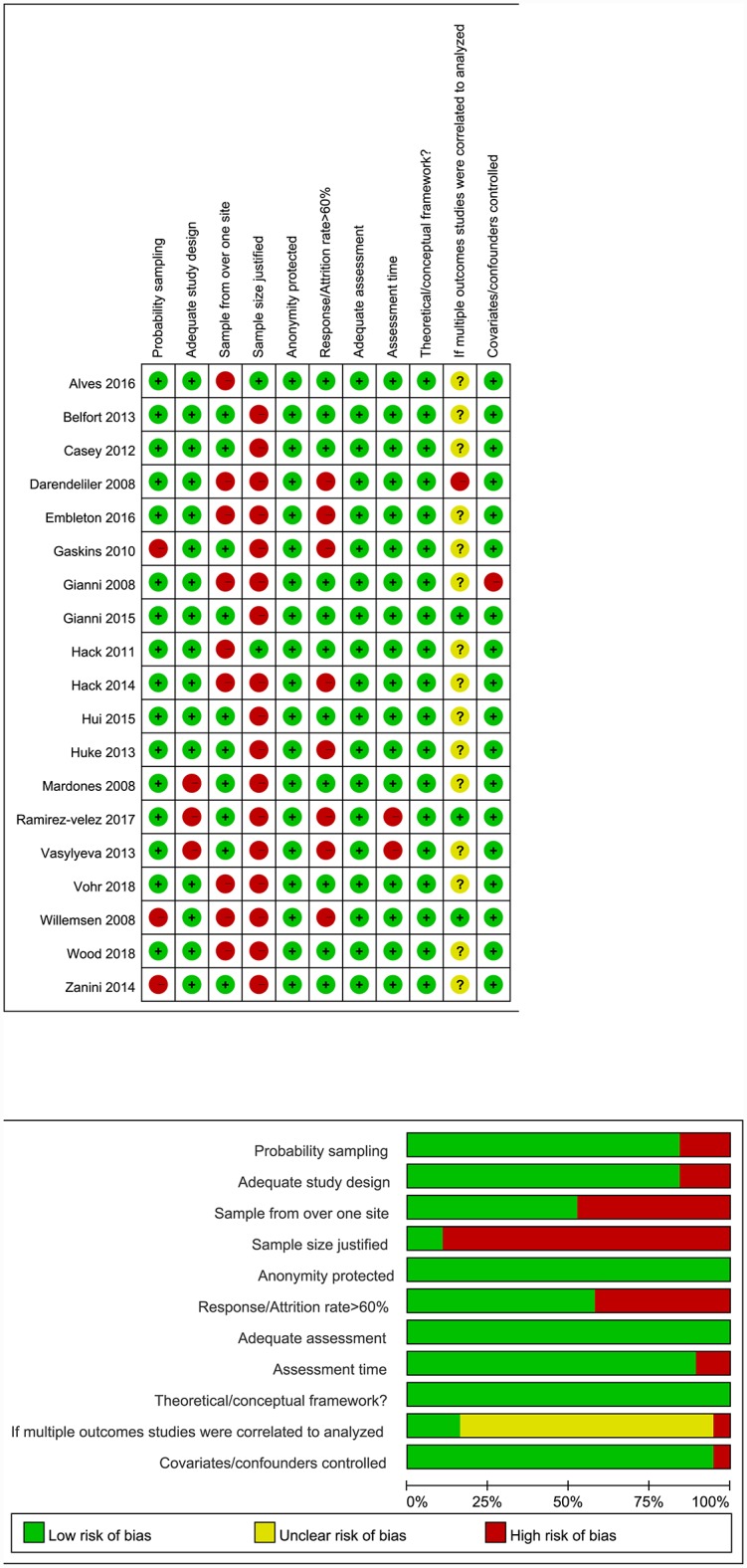
Quality assessment of studies included.

The quality of all the studies included is of moderate level; nine studies received scores that indicated high quality (Fig 2). The studies included adequate assessment, theoretical frameworks, and methods to protect anonymity. However, only two of the 19 studies justified their sample size, and 11studies had a response rate above 60%, ninestudies collected samples from more than one site, and 18 studies addressed controlled confounders.

### Research question 1: What is the impact of GA on the risk of childhood obesity? (preterm versus term infants)

#### Childhood obesity and preterm status

**Fig 3:**
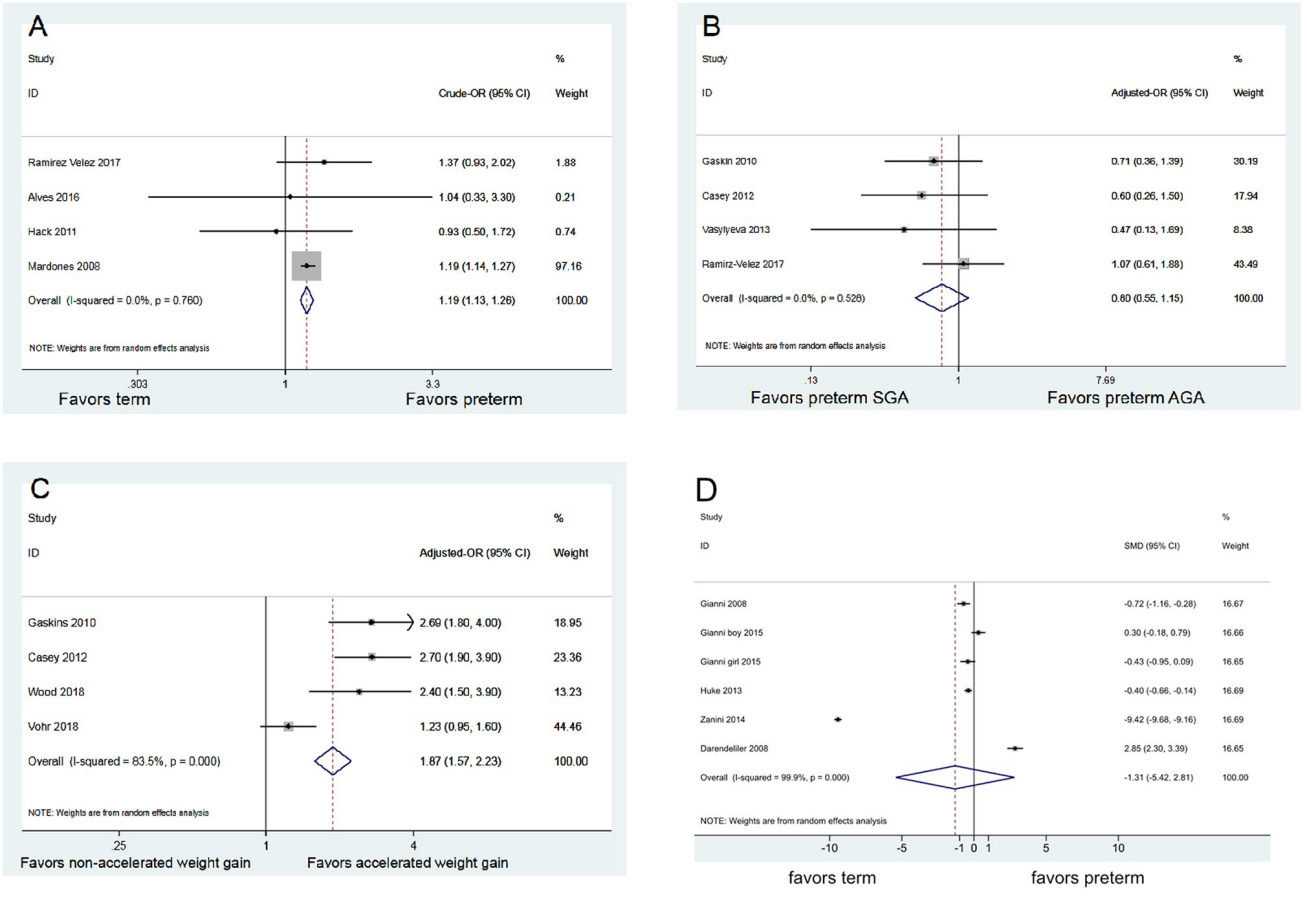
Summary effects. (A) Impact of preterm status on childhood obesity (B) Impact of preterm SGA (vs. preterm AGA) on childhood obesity (C) Impact of accelerated weight gain on childhood obesity (D) Association between childhood fat mass index and preterm status.

In our analysis, childhood obesity was a dichotomous outcome (yes/no) which is defined by whether the children’s BMI measures were ≥ 95th percentile (based on age- and sex-specificc growth standards). Four studies (mean quality assessment: 8 points) enrolled 156,439 children reported rates on obesity. The result of meta-analysis revealed that preterm infants had a greater likelihood of childhood obesity compared to term infants (crude OR = 1.19; 95% CI [1.13, 1.26], *p* < 0.001; Fig 3A) [14, 39–41).

#### Childhood fat mass index and preterm status

Five studies, with a mean quality assessment of 8 points, consisted of 668 childrenprovided data on the fat mass index and were included in the meta-analysis. No statistical difference was found between preterm and term infants in fat mass index assessed at age 4 to 7 (standardized mean difference (SMD -1.31; 95% CI [-5.42, 2.81]; *p* = 0.535; Fig 3D) [42–46]. Separate subgroup analyses based on whether the study outcomes were measured by dual-energy x-ray absorptiometry (DEXA) still revealed no significance between preterm and term andin childhood fat mass index (SMD -2.431; 95% CI [-10.198, 5.336]; *p* = 0.54 [42, 45,46])(S1 Fig) versus (SMD -0.195; 95% CI [-0.635, 0.246]; *p* = 0.387) ([43,44]) (S2 Fig).

#### Childhood the percentage body fat and preterm status

Two studies (mean quality assessment: 8) with 1,970 children provided data on the percentage of body fat and were included in the meta-analysis. No significant difference was observed between preterm and term children at age 4 to 7 (SMD -3.057; 95% CI [-8.736, 2.621]; *p* = 0.291) [44, 45]).

#### Childhood trunk fat index and preterm status

Two studies (mean quality assessment: 7) with totally 274 children provided data on trunk fat index and were included in the meta-analysis.No significance difference was found between preterm and term children at age 4 to 7 (SMD 1.033; 95% CI [-1.646, 3.712]; *p* = 0.450) [42,46]).

#### Childhood body composition indices and preterm status (no data synthesis)

Three studies compared childhood body compositions between preterm and term infants. These outcomes could not be pool-estimated because of their un-uniformed outcome measures. We qualitatively reviewed these studies as followed. Willemsen et al. (2008) reported data of 144 children and concluded that preterm SGA children had a lower percentage of body fat than term infants with SGA assessed at age 6 to 7 [47]. Assessments of abdominal fat included total abdominal adipose tissue (TAAT), percent of ratio of intra-abdominal adipose tissue (IAAT)/TAAT (% IAAT), trunk fat/total fat, waist-hip ratio, and waist-height ratio. Huke 2013 and Willemsen 2008 found that preterm infants had a lower risk of central obesity than term infants in TAAT and trunk fat assessments. However, no significances were found in % IAAT and ratio of trunk fat/total fat [44, 47]. Hui 2015 studied 7,169 children and found that late premature infants (defined as 34 to 36 weeks of gestation) had a greater waist-hip ratio z-score (β = 0.16; 95% CI [0.03, 0.29]) and waist-height ratio z-score (β = 0.27; 95% CI [0.14, 0.40]) than term infants in adolescence [48]. (Table 2).

**Table 2:** Comparison of childhood body composition indices between preterm and term infants.

	Body Composition	Measured by	Age at evaluation	Preterm (n)	Term (n)	Difference (95%CI)	*p*
Willemsen 2008 [47]	% body fat SDS_age_	DEXA	mean6.8y	-1.2 (0.8) (n = 51)	-0.6 (0.9) (n = 93)		*p* = 0.011
				273 (166–451) (n = 53)	609 (338–936) (n = 93)		*p* = 0.043
	trunk fat(g)						*p* = 0.795
	trunk fat/total fat						
				0.33 (0.05) (n = 53)	0.34 (0.05) (n = 93)		
Huke 2013 [44]	TAAT (cm^3^)	MRI	5–7 y	72.1±33.8 (n = 68)	87±55.8 (n = 86)	-14.93 (-29.31,-0.53)	*p* = 0.04
	IAAT (cm^3^)			19.9±7.7 (n = 68)	21.7±9.0 (n = 86)	-1.87 (-4.53,0.78)	*p* = 0.17
	%IAAT (%)			30±9 (n = 68)	28±9 (n = 86)	1.8 (-1.18,4.78)	*p* = 0.23
Hui 2015 [48]	waist-hip ratio z-score	z-score	14 y	0.16 (95% CI [0.03, 0.29]) (preterm vs. term)			
	waist-height ratio z-score			0.27 (95% CI [0.14, 0.40]) (preterm vs term)			

Abbreviations: CI, confidence interval; SDS, standard deviation score; y, year; TAAT, total abdominal adipose tissue; IAAT, intra-abdominal adipose tissue; % IAAT, IAAT/TAAT; MRI, magnetic resonance imaging.

### Research question 2: In preterm infants, is SGA associated with a greater risk of childhood obesity than AGA infants?

Four studies (mean quality assessment: 7) contained 3,655 children provided adjusted ORs for the associations between SGA status and childhood obesity. The result of meta-analysis revealed no significant difference on childhood obesity between SGA and AGA infants (adjusted OR = 0.80; 95% CI [0.55, 1.15]; *p* = 0.226; Fig 3B) [9, 11, 14, 49]. In addition,two studies not included in the data synthesis(due to ununiformed outcome definition) were also reviewed.Darendeliler *et al*. reported no significant differences between preterm SGA and preterm AGA children with respect to fat mass index and truck fat index (central obesity) at 4 to 6 years of age [46], whereas Gianni *et al*. found that being SGA positively affected trunk fat mass (*r*^*2*^ = 0.37; *p* < 0.05) [42].

### Research question 3: Does accelerated weight gain in preterm infants increase the risk of childhood obesity?

Fourstudies (mean quality assessment: 8) consisted of 2,129 participants and provided adjusted odds ratios on the association between accelerated weight gain and childhood obesity at age 8–11 among preterm infants. Accelerated weight gain was defined as weight gain velocity during the first two years from birth (the details are summarized in Table 1).The result of meta-analysis revealed that accelerated weight gain is significantly associated with an increased odds of childhood obesity (adjusted OR = 1.87; 95% CI [1.57, 2.23]; *p* < 0.001; Fig 3C)[9, 49–51]. wo other studies not included in the data synthesis(due to ununiformed outcome definition) were also reviewed: Belfort *et al*. reported that preterm infant BMI gain in three intervals (term to 4 months, 4 to 12 months, 12 to 18 months) was associated with a higher odds of overweight/obesity at 8 years of age (term to 4 months: OR = 1.36 per additional z-score BMI gain, 95% CI [1.14, 1.62]; 4 to 12 months: OR = 1.66; 95% CI [1.33, 2.06]; 12 to 18 months: OR = 2.00; 95% CI [1.53, 2.61] [52]. Embleton *et al*. also reported strong associations between more rapid childhood weight gain after 1 year of age and subsequent general and central obesity (higher fat mass percentage, fat mass index, and waist circumference) in adolescents born preterm (coefficient 5.03; 95% CI [3.74, 6.32]; coefficient 1.74, 95% CI [1.35, 2.13]; coefficient 5.89, 95% CI [4.28, 7.50]) [21].

## Discussion

The purpose of this study was to determine the association between preterm birth and the risk of childhood and adolescent obesity and the impact of accelerated weight gain in preterm infants on the risk of childhood obesity. Our results revealed that premature infants had a greater likelihood of childhood obesity at age 6 to 16than infants born at term.In addition, accelerated weight gain in preterm infants significantly increased the likelihood of childhood obesity at age 8 to 11. No significant difference on childhood obesity risk between SGA and AGA preterm infants was found.

We found that preterm infants had a greater likelihoodof childhood obesity than the term (1.2 times), as determined by BMI percentile, than term infants. However, no differences were found when outcomes measured by other indices of adiposity. Inconsistent findings on lower body fat percentage, total abdominal adipose tissue, trunk fat mass, higher waist-hip z score, and waist-height z score were found in three studies in our systematic review.

We found that preterm infants had a higher risk of general obesity, as measured by BMI, than infants born at term. This is consistent with several earlier studies reproted that preterm infants had an increased risk of childhood obesity, central adiposity, and metabolic syndrome [53–56]. Few studies have examined the potential link between prematurity, as determined by GA, and the risk of childhood obesity, and their results have been inconsistent. For example, one study found a lower risk in preterm infants [57] whereas another two studies found a higher risk [58, 59]. In addition, other studies have reported that preterm infants might be associated with higher childhood body fat as compared to term infants [54, 60,61]. BMI has been used as a measure of overall obesity and is routinely applied to estimate body fat, not only in epidemiological studies, but also in clinical practice. Although BMI is not a very precise measure of adiposity in individuals, and is considered an imperfect measure of body fatness [62–64] as it cannot discriminate between lean mass and fat mass, excess body fat mass is the hallmark of obesity and related to morbidity and mortality. Moreover, BMI explains from 20%-75% of the variability in body fat composition in children [62,63,65], and the error made by BMI in estimating body fat is usually acceptable at the population level. Thus, BMI has been the most widely recognized surrogate of obesity, albeit not a valid parameter for assessment of the distribution of body fat. The heterogeneity in measures of body composition could be attributed in part to differences in the methods used as well as the timing of measurements.

We found that accelerated weight gain was a critical risk factor for childhood obesity. In preterm infants (birth to 1 year), accelerated weight gain significantly increased the risk (2.69 times) of obesity in middle childhood (8 to 11 years old). This finding is consistent with other systematic reviews that focused on term infants. This suggests that rapid weight gain during infancy is associated with a two-to-fourfold increased risk of obesity [22, 66–68]. Although the mechanism by which rapid weight gain leads to childhood obesity remains unclear, several hypotheses have been proposed for an increased risk of obesity, including perinatal programming [69], genetic factors, nutritional factors, parental feeding practices, and others [18, 68, 70–72]. A proposal has been advanced, supported by Barker’s hypothesis and animal studies, that epigenetic programming may play an important role in the development of obesity [73, 74]. Elks *et al*. [75] observed that adult obesity risk alleles contribute to early infancy weight gain and growth. Yeung suggests that β3 adrenoceptor polymorphism-mediated lipolysis and insulin resistance secondary to hyperinsulinemia could increase the accretion of visceral adiposity [18]. Some researchers have suggested that leptin may play a role in the regulation of weight gain in infancy, and reported an association between higher leptin at age 3 and greater weight gain and adiposity in later childhood [68]. Future studies should examine the role of epigenetic programming among preterm infants and its association with rapid weight gain.

In addition to genetic factors, nutritional factors such as breastfeeding in rapid weight gain infants have been associated with a lower percentage of body fat later in life [76]. Evidence suggests that human milk is high in adiponectin, an insulin-sensitizing and anti-inflammatory molecule, which affects infants’ weight trajectories during the first 2 years of life [77]. Formula or mixed feeding has been associated with greater weight gain from birth to 1 to 3 years of age and a higher BMI later in life (1 to 5 years) [78]. Higher nutrient intake and growth acceleration in the immediate postnatal period have also been associated with later metabolic risks in infants born preterm [79, 80]. Breastfeeding during the first year of life and the slow introduction of baby food may have life-long benefits for preterm babies and be a practical approach to prevent obesity [81].

From a neurodevelopmental perspective, aggressive nutritional interventions for catch-up growth in preterm infants would appear to be justified. However, early weight gain may contribute to later obesity without cognitive benefits [52]. Long-term metabolic syndrome associated with catch-up growth poses an apparent dilemma in nutritional management, especially for vulnerable preterm infants. Early nutritional consultation and healthy catch-up such as maintaining a normal growth trajectory are key steps to prevent obesity. Future studies should investigate the role of weight gain in both physiological and cognitive development in preterm infants and identify an appropriate weight gain trajectory for this population.

Our meta-analysis found no difference in the risk of obesity in preterm infants by weight status alone (SGA vs. AGA). These findings are consistent with those of two studies [46, 82] that found no differences in truncal fat and BMI between preterm AGA and SGA infants. These findings, however, are inconclusive. One study found that SGA infants had a higher risk [83], while other studies have suggested a lower risk for childhood obesity [84, 85]. Data are scarce on the subsequent general obesity in preterm SGA infants compared with preterm AGA infants during middle childhood. Future studies should focus on preterm infants with SGA and identify the mechanism of obesity in this population.

### Limitations and implications for further research

To the best of our knowledge, this is the first meta-analysis and systematic review to examine the risk of childhood obesity in preterm infants. However, there are several limitations.First, the number of studies included for each individual data synthesis is relatively limited. Given a meta-analysis with a small sample size may increase the risk for overestimation or underestimation of the effect size, results shoud be interpreted with caution[86, 88]. Nevertheless, we have analyzed all evidence available under a rigorously selected criteria. Second, although most of the studies were of moderate to high quality, risk factors such as complications atbirth, parental education level and, socio-economic status, or parental obesity status may have not been adjusted by all the studies, which may possibly introduce bias into the results.

Advances in perinatal care have led to remarkable improvements in the survival rates of preterm infants born between 22 and 25 weeks of gestation [89]. The degree of prematurity and growth influences later outcomes. The etiology of obesity is multifactorial, and is influenced by complex interactions between genetic, environmental, and psychosocial factors. Furthermore, the observational studies had confounding factors such as socioeconomic status and environmental factors, maternal BMI, education, ethnicity, lifestyle (particularly physical activity), and nutritional factors. More longitudinal research and prospective controlled trials using detailed standardized measurements in early life and later childhood of preterm infants are needed. By identifying the underlying mechanism that leads to obesity, researchers may be able to develop effective therapies to decrease obesity-related comorbidities.

## Conclusion

Preterm infants are prone to developing deleterious, long-term health outcomes, especially obesity and metabolic problems. In our review, preterm children had a higher chance of childhood obesity. Accelerated weight gain in preterm children, especially in infancy, was one of the contributors to obesity in later life. Establishing optimal growth patterns or healthy catch-up is needed to minimize the long-term risk of obesity. Carefully monitoring weight during infancy and childhood and timely referral to health care providers for lifestyle interventions and dietary recommendations may decrease the risk of obesity-related comorbidities.

## Supporting information

S1 Checklist PRISMA 2009 checklist. (DOCX)

S1 Fig (TIF)

S2 Fig (TIF)
