# Correction: Accelerated weight gain, prematurity, and the risk of childhood obesity: A meta-analysis and systematic review

**DOI:** 10.1371/journal.pone.0298556

**Published:** 2024-02-05

**Authors:** Mei-Chen Ou-Yang, Yao Sun, Melissa Liebowitz, Chih-Cheng Chen, Min-Lin Fang, Weiwei Dai, Tang-Wei Chuang, Jyu-Lin Chen

After this article [1] was published, the authors identified errors in Fig 3. In Fig 3B, the x-axis labels “Favors preterm SGA” and “Favors preterm AGA” are swapped. The left label should be “Favors preterm AGA” and the right label should be “Favors preterm SGA”. Also in Fig 3B, there is an error in the reported aOR (95% CI) for Gaskin 2010. This error also resulted in incorrect % weight values, the I-squared statistic and p value, and aOR (95% CI) overall. The authors provide a corrected Fig 3 here. In the second sentence of the Research question 2 subsection of the Results, the correct sentence is: “The result of meta-analysis revealed no significant difference on childhood obesity between SGA and AGA infants (adjusted OR = 1.03; 95% CI [0.69, 1.53]; p = 0.107; Fig 3B)”

**Fig 3:**
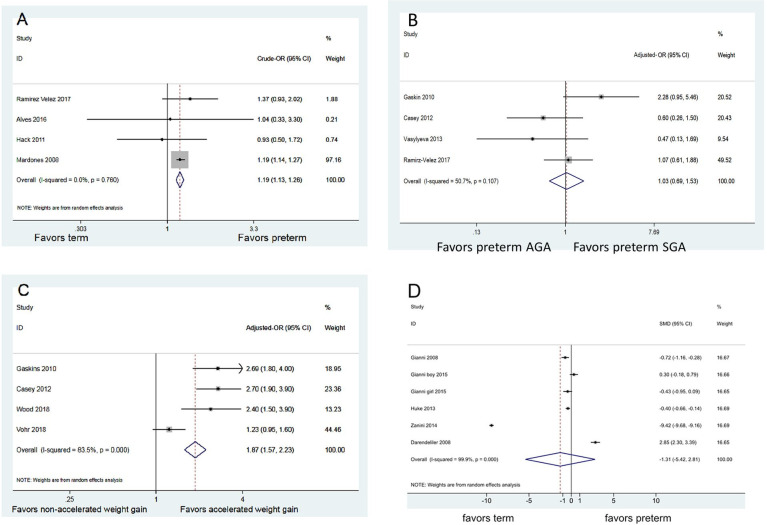
Summary effects. (A) Association between preterm status and childhood obesity (B) Association between preterm SGA (vs. preterm AGA) and childhood obesity (C) Association between accelerated weight gain and childhood obesity (D) Association between childhood fat mass index and preterm status.

In addition, after publication of this article [1], concerns were raised about the use of the term “impact”, as this incorrectly implies a causal relationship. Therefore, “impact of…on” is corrected throughout the article to “association between…and”.

The authors apologize for the errors in the published article, which do not affect the Conclusions.

Some concerns were also raised about possible biases in the statistical analyses.

Concerns were raised that confounding effects may have introduced bias and may not have been adequately discussed. The authors provide additional information to Table 1 here about the covariates/confounders that were adjusted and reported in each original study selected.

**Table 1:** Characteristics of the studies included.

First author, year	Study design /country	Total number of children	Number of preterm, term, SGA / AGA, and gestational age	Age of OBE evaluation (years)	Indices for OBE	Covariates /confounders for adjustment	Main finding
Alves, 2016 [39]	Retrospective, Brazil	134	67 Preterm (mean GA 33.2 wk); SGA: 30; 67 Term	10–13	BMI >97th percentile;	age, sex, ethnicity, birth weight SDS, birth length SDS, BMI SDS and height SDS	OBE, preterm vs. term: 6 (14.3%) vs. 6 (12.2%), p = 1.00
Belfort 2013 [52]	Retrospective, USA	945	945 Preterm (median GA 33.26 wk); SGA: 327	8–18	Z-score for corrected age	age, sex, gestational age, maternal age, education, smoking in pregnancy, and annual household income	Associations with overweight/OBE: BMI z-score change at 4 months: adjusted OR = 1.36 (1,14–1.62) BMI z-score change at 4–12 months: adjusted OR = 1.66 (1.33, 2.06) BMI z-score change at 12–18 months: adjusted OR = 2.00 (1.53, 2.61)
Casey, 2012 [49]	Prospective USA	686	686 Preterm (mean GA 33 wk)	8	BMI for age-sex ≥ 95^th^ percentile	gestation	Associations with OBE: Rapid weight gain (weight gain velocity 100g/mo, from birth to 12-mo): adjusted OR = 2.7, 95% CI [1.9, 3.9] SGA status: adjusted OR = 0.6, 95% CI [0.26, 1.5].
Darendelil, 2008 [46]	Prospective, Turkey	179	93 Preterm (≤ 37 wk); AGA / SGA: 63 / 30; 86 Term; AGA / SGA: 44 / 42	4.7	Fat mass index	birth weight	Preterm AGA vs term AGA had similar fat mass index and trunk fat index. Fat mass index: 3.6±0.4 vs. 2.7±0.1; trunk fat index:1.5±0.2 vs. 1.1± 0.1. Preterm SGA vs. preterm AGA had similar fat mass index and trunk fat index. (2.9±0.5 vs. 3.6±0.4; 1.2±0.3 vs. 1.5±0.2).
Embleton, 2016 [21]	Prospective, UK	98	98 Preterm (mean GA 30.8 wk)	11.5	% Body Fat; Fat mass index; Waist circumference	gestation, birthweight SDS, requirement for mechanical ventilation, sex, and current age at follow-up and pubertal status	Adolescent height and weight SDS did not differ between rapid weight gain or not (0.01±0.92 and 0.3±1.2, respectively). (Rapid weight gain: weight z score change > 0.67 from term to 12 wk, n = 24) Rapid weight gain after 1 year of age was associated with subsequent higher % fat mass, fat mass index and waist circumference (coefficient: 5.03, 95% CI [3.74, 6.32]; 1.74, 95% CI [1.35, 2.13]; 5.89, 95% CI [4.28, 7.50]).
Gaskins, 2010 [9]	Prospective, USA	312	312 Preterm (≤ 32 wk:115, 32–36 wk: 197); SGA/AGA: 67/245	11	BMI for age-sex >95^th^ percentile	gestational age	Associations with OBE: Rapid weight gain (weight gain velocity g/mo, from birth to 12-mo): adjusted OR = 2.69; 95% CI [1.80, 4.00] SGA status: adjusted OR = 2.28; 95% CI [0.95, 5.46].
Gianni, 2008[42]	Prospective, Italy	95	45 Preterm (<34 wk; mean GA 30.5±1.9 wk); 40 Term (mean GA 39.1±1.3 wk)	4.8–6.6	Fat mass index	gestational age	Fat mass index (kg/m^2^) was lower in preterm (2.76±1.16 vs. 3.76±1.58, *p* < 0.05); Trunk fat index (kg/m^2^) was not significant (PT vs. FT, 0.94±0.73 vs. 1.18±0.72); Preterm SGA positively affected trunk fat mass content (r^2^ = 0.37, *p* < 0.05).
Gianni, 2015 [43]	Prospective, Italy	124	63 Preterm (< 32 wk); 61 Term	5	% Body fat; Fat mass index	gestational age	% Body fat and fat mass index were similar in both groups. % Body fat: 20.4±5 (boy PT) vs 17.7 ± 5.5 (boy FT); 20.5±5.1 (girl PT) vs. 23.1±4.7 (girl FT). Fat mass index: 3.1±0.9 (boy PT) vs. 2.9±0.1 (boy FT); 3.2±1.1 (girl PT) vs. 3.7±1.2 (girl FT).
Hack, 2011 [40]; 2014 [53]	Prospective, USA	259	146 Preterm (mean GA 26.5 ± 2); 113 Term	14	BMI for age-sex ≥ 95^th^ percentile	SES, race, and sex	OBE, preterm vs. term: 28 vs. 23
Hui, 2015[48]	Prospective, Hong Kong	7169	295 Preterm (mean GA 35.4 wk); 6874 Term	14	BMI z score; WHR z-score; WHtR z-score;	Model 1: adjusted for sex, highest parents’ education attainment, mother’s place of birth, pregnancy characteristics including gestational diabetes, preeclampsia, and maternal smoking, presence of birth defects, and age (years) at measurements (except for BMI z-score). Model 2: additionally adjusted for accelerated growth from birth to 12 months.	Preterm had greater WHR z-score (β = 0.16, 95% CI [.03, 0.29]) and WHtR z-score (β = 0.27, 95% CI [0.14, 0.40]) compared with term infants.
Huke, 2013[44]	Retrospective, Germany	236	116 Preterm (≤ 33 wk; mean GA 29.8 ± 2.6 wk); 120 Term	5–7	% Body fat; Fat mass index; Waist-hip circumferences; Adipose tissue by MRI	NA	Waist-hip circumferences similar in both group (0.97 vs. 0.96 in preterm and term group). % Body fat, fat mass index were lower than term group (18% vs. 21%, *p* = 0.0022; 2.82 ± 1.4 vs. 3.36 ± 1.32 kg/m2, *p* = 0.028) TAAT(cm^3^): preterm vs. term: 72.1 ± 33.8 vs.87 ± 55.8, *p* = 0.04% IAAT (%): preterm vs. term: 30 ± 9 vs. 28 ± 9, *p* = 0.23.
Mardones, 2008 [41]	Retrospective, Chile	153536	17574 Preterm 135962 Term	6–8	BMI for age-sex ≥ 95^th^ percentile	sex, GA, BW, BL, education, and height for age at 6–8 years age.	OBE, Preterm vs. term: 17.53% vs. 18.05%, p = 0.088
Ramirez-Velez, 2017 [14]	Retrospective, Colombia	2510	1092 Preterm (< 37 wk); SGA / AGA: 249 / 843; 1418 Term; SGA / AGA: 260 / 1158	11–14 Mean age:13.2	BMI for age-sex ≥ 95^th^ percentile	age, pubertal stage, and weight status by gender.	Risks of OBE were not significant different in preterm vs term, adjusted OR = 1.373, 95%CI [0.93, 2.02]) (preterm vs. term: n = 54 vs. 55) SGA status was significantly associated with OBE, adjusted OR = 1.07, 95% CI [0.42, 1.03])
Vasylyeva, 2013 [11]	Retrospective, USA	147	147 Preterm (≤ 37 wk); SGA/AGA: 23/124	10–20	BMI for age-sex ≥ 95^th^ percentile	age	Associations with OBE: SGA status: adjusted OR = 0.47, 95% CI [0.13, 1.69].
Vohr, 2018 [50]	Prospective, USA	388	388 Preterm	6–7	BMI for age-sex ≥ 95^th^ percentile	NA	Rapid weight gain (weight gain velocity kg/yr from birth to 18–22 months) was not significantly associated with OBE, adjusted RR = 1.23, % 95CI [0.95, 1.60], p<0.123
Willemsen, 2008 [47]	Retrospective, Netherlands	144	51 Preterm (< 36 wk, all SGA); 93 Term (all SGA)	6.8	% Body fat SDS; Trunk fat/total fat	age, sex, ethnicity, birth weight SDS, birth length SDS, and total body weight	PT SGA had lower body fat SDS than term SGA (-1.2 (0.8) vs. -0.6(0.9), Similar trunk fat/total fat 0.33 (0.05) vs. 0.34 (0.05).
Wood, 2018 [51]	Prospective, USA	743	743 Preterm	10	BMI for age-sex ≥ 95^th^ percentile	NA	Rapid weight gain (top quartile weight gain from birth to 12 months) was significantly associated with OBE, adjusted OR = 2.4, 95% CI [1.5–3.9].
Zanini, 2014 [45]	Prospective, Brasil	1734	416 Preterm (< 37 wk) 1318 Term	6.7	% Body fat; Fat mass index	birth weight and height	Lower % body fat and fat mass index were found in preterm. % Body fat: PT vs. FT: 17.61±0.53 vs. 21.05±0.16; fat mass index: PT (*n* = 403) vs. FT (*n* = 2643), 3.18±0.14 vs. 3.83±0.05.

**Abbreviations:** *n*, sample size; SGA, small for gestational age; AGA, appropriate for gestational age; BMI, body mass index; OBE, obesity; OR, odds ratio; CI, confidence interval; GA, gestational age; f/u, follow-up; y/o, year old; DEXA, dual-energy x-ray absorptiometry; SDS, standard deviation score; BIA, bioelectrical impedance analysis; MRI, magnetic resonance imaging; TAAT, total abdominal adipose tissue; PT, preterm; FT, full term; %IAAT, intra-abdominal adipose tissue/total abdominal adipose tissue; WHR, waist-hip ratio; WHtR, waist-height ratio; wk, week; socioeconomic status (SES); BW, birth weight; BL, birth length

Concerns were also raised about the lack of sensitivity analyses included in the study. The authors provide an additional S1 Table to summarize the sensitivity analysis using the “leave-one-out” approach, below. The authors provide the following clarifications discussing the results of the sensitivity analysis, which does not affect the original conclusions.

It was noted that in the meta-analysis assessing the association between preterm status and childhood obesity (Fig 3A), one study (Mardones et al. 2008) [2] contributed 97% of the data. The authors clarify here that during the sensitivity analysis, as shown in S1 Table, the pooled estimate shifted to non-significant (OR = 1.213, 95%CI: 0.884, 1.663, p = 0.232) upon excluding Mardones et al. (S1 Table). Accordingly, readers are advised to exercise caution when interpreting the conclusion of this meta-analysis on the difference between preterm vs term infants in association with childhood obesity, as the pooled estimate is substantially influenced by the study of Mardones et al.

It was also noted that in the meta-analysis assessing the association between preterm SGA (vs. AGA) and childhood obesity (Fig 3B), one study (Ramírez-Vélez et al.) [3] that controls for later weight contributed the most data to the pooled estimate. The authors clarify here that during the sensitivity analysis, as shown in S1 Table, the pooled estimate remained similar to the original estimate (OR = 0.932, 95%CI: 0.416; 2.089, p = 0.865) upon excluding Ramírez-Vélez et al., suggesting the meta-analysis outcome is robust (S1 Table).

Finally, it was noted there was a high degree of heterogeneity among the included estimates for the meta-analysis assessing the association between accelerated weight gain and childhood obesity (Fig 3C). The authors clarify here that during the sensitivity analysis, as shown in S1 Table, the heterogeneity reduced upon excluding the study by Vohr et al. [4]. In Fig 3D, substantial heterogeneity was also noted across the studies. Yet, upon excluding individual studies during sensitivity analysis, there was no reduction in the observed heterogeneity (S1 Table).

Concerns were also raised that seven studies that report BMIs of both preterm and term-born children [5–11] were excluded from the meta-analysis comparing childhood obesity risk between preterm and term infant (Fig 3A), without sufficient justification. The authors clarify here that these studies were excluded from analysis due to specific reasons. Forsum et al. (2019) [5] only presented data comparing BMI between full-term boys and girls, lacking a comparison of childhood obesity status between preterm and term-born infants. Other studies [6–11] either reported BMI data in continuous values or presented z-scores incompatible with studies using dichotomized obesity status in the present meta-analysis. The authors wish to clarify that the criterion of childhood obesity (i.e., age and sex-specific BMI > = 95^th^ percentile) was chosen based on its frequent usage in the included articles. This approach was specifically selected to mitigate potential confounding effects related to age and gender when studying pediatric BMI.

In addition, the authors clarify that no test for publication bias was performed since none of the meta-analyses performed included ten or more studies.

A member of the Editorial Board reviewed the article and concerns raised, and advised that additional information and clarifications to the statistical analyses were required to support the study conclusions. A member of the Statistical Advisory Group reviewed the article and concerns raised, and advised that the corrections and clarifications above address the concerns and support the results and conclusions reported in the article; they confirmed that following sensitivity analysis, the original findings still stand.

## Supporting information

S1 Table Sensitivity analysis. (DOCX)
