# Correction: Correction: Accelerated weight gain, prematurity, and the risk of childhood obesity: A meta-analysis and systematic review

**DOI:** 10.1371/journal.pone.0299066

**Published:** 2024-02-13

**Authors:** The PLOS ONE Staff

## Notice of republication

This article was republished on February 6^th^, 2024, to correct an error in the title. The publisher apologizes for the error. Please download this article again to view the correct version.
