# Supplementary figures and images for: Accelerated weight gain, prematurity, and the risk of childhood obesity: A meta-analysis and systematic review
Source: PLoS One. 2020 May 5;15(5):e0232238. doi: 10.1371/journal.pone.0232238 (PMC7199955; doi:10.1371/journal.pone.0232238)

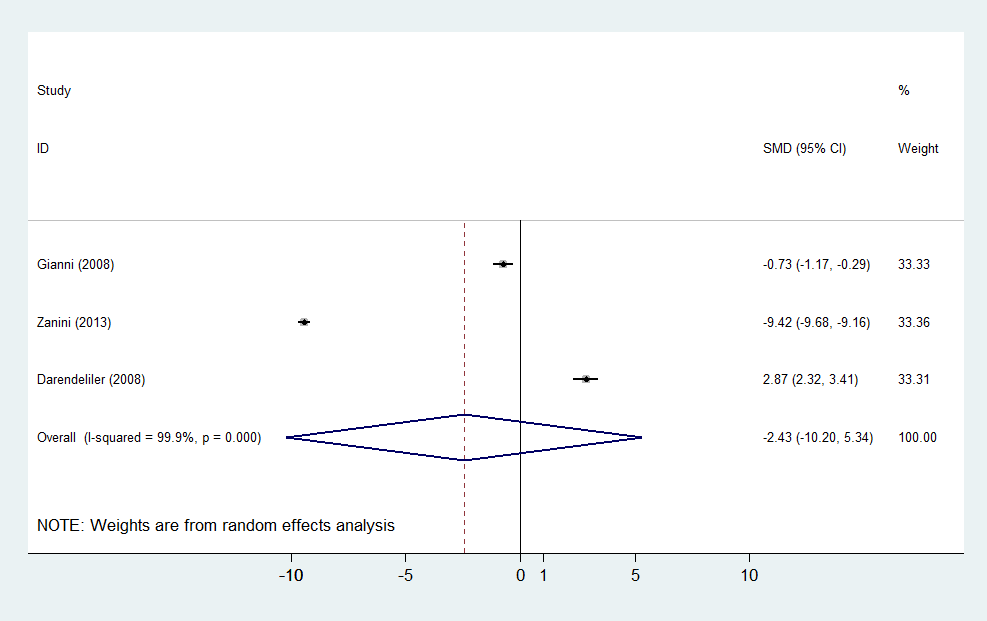

Supplement: S1 Fig — (TIF) [file pone.0232238.s002.tif]

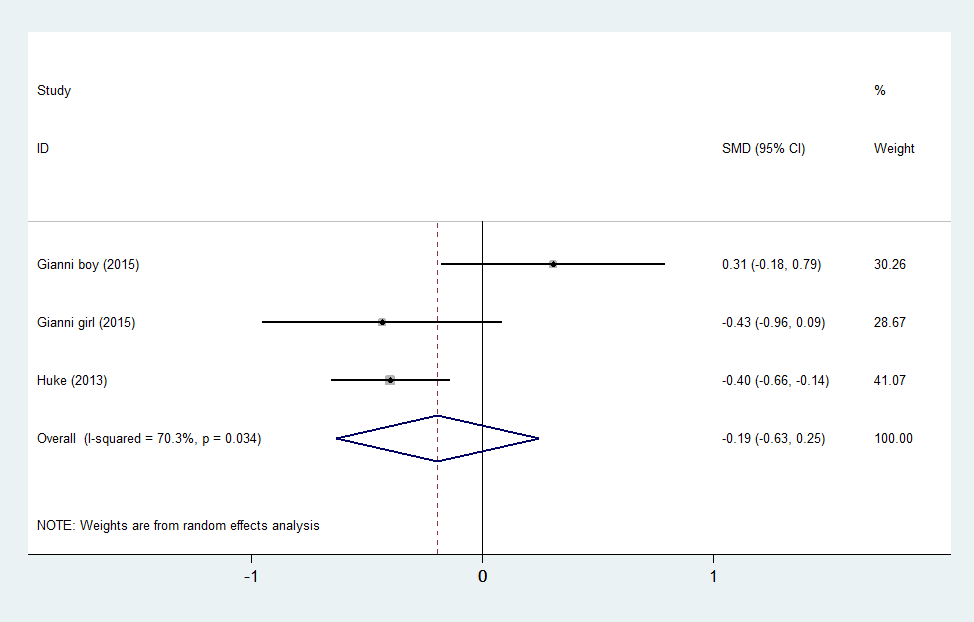

Supplement: S2 Fig — (TIF) [file pone.0232238.s003.tif]
