# Supplementary material for: Correction: Accelerated weight gain, prematurity, and the risk of childhood obesity: A meta-analysis and systematic review
Source: PLoS One. 2024 Feb 5;19(2):e0298556. doi: 10.1371/journal.pone.0298556 (PMC10843033; doi:10.1371/journal.pone.0298556)
Supplement: S1 Table — (DOCX) [file pone.0298556.s001.docx]

**S1 Table. Sensitivity analysis**

| Study left out | OR/SMD | 95% CI | p-value | tau^2^ | I^2^ |
| --- | --- | --- | --- | --- | --- |
| **Preterm vs term** |  |  |  |  |  |
| Ramirez Velez 2017 | 1.189 | [1.146; 1.235] | < 0.001 | 0 | 0% |
| Alves 2016 | 1.191 | [1.147; 1.237] | < 0.001 | 0 | 0% |
| Hack 2011 | 1.192 | [1.148; 1.238] | < 0.001 | 0 | 0% |
| Mardones 2008 | 1.213 | [0.884; 1.663] | 0.232 | 0 | 0% |
| **Preterm SGA vs. preterm AGA** |  |  |  |  |  |
| Gaskin 2010 | 0.836 | [0.536; 1.303] | 0.428 | 0 | 3.10% |
| Casey 2012 | 1.178 | [0.756; 1.837] | 0.469 | 0 | 53.20% |
| Vasylyeva 2013 | 1.117 | [0.715; 1.747] | 0.627 | 0.02 | 55.70% |
| Ramirz-Velez 2017 | 0.932 | [0.416; 2.089] | 0.865 | 0.25 | 66.90% |
| **Accelerated weight gain vs non-accelerated weight gain** | | |  |  |  |
| Gaskins 2010 | 1.936 | [1.271; 2.948] | 0.002 | 0.10 | 86.00% |
| Casey 2012 | 1.920 | [1.261; 2.924] | 0.002 | 0.10 | 84.60% |
| Wood 2018 | 2.025 | [1.310; 3.133] | 0.002 | 0.12 | 88.20% |
| Vohr 2018 | 2.622 | [2.077; 3.311] | < 0.001 | 0 | 0% |
| **Preterm vs term (fat mass index)** | | | | | |
| Gianni 2008 | -1.423 | [-7.224; 4.377] | 0.533 | 17.43 | 99.90% |
| Gianni 2015 | -1.628 | [-7.338; 4.082] | 0.473 | 16.89 | 99.90% |
| Gianni 2015 | -1.482 | [-7.265; 4.301] | 0.516 | 17.32 | 99.90% |
| Huke 2013 | -1.489 | [-7.271; 4.293] | 0.514 | 17.32 | 99.90% |
| Zanini 2014 | 0.312 | [-1.495; 2.118] | 0.657 | 1.62 | 96.90% |
| Darendeliler 2008 | -2.136 | [-7.220; 2.948] | 0.308 | 13.39 | 99.90% |

OR, odds ratio; SMD, standardized mean difference.
